# Laparoscopic Mesh-Salvage Fenestration for Chronic Symptomatic Seroma after Rives–Stoppa Incisional Hernia Repair: A Case Report

**DOI:** 10.70352/scrj.cr.26-0510

**Published:** 2026-09-05

**Authors:** Masanori Sato

**Affiliations:** Division of General (Endoscopic) Surgery, Department of Surgery 1, Hamamatsu University School of Medicine, Hamamatsu, Shizuoka, Japan

**Keywords:** chronic seroma, incisional hernia, laparoscopic fenestration, mesh salvage, retromuscular mesh

## Abstract

**INTRODUCTION:**

Chronic encapsulated seroma after retromuscular incisional hernia repair can be difficult to manage, particularly in symptomatic patients without evidence of mesh infection. Open anterior capsulectomy may require wide mesh exposure and carries potential risks of mesh infection and recurrent incisional hernia. Herein, I report a case of a chronic symptomatic seroma successfully managed by laparoscopic mesh-salvage fenestration.

**CASE PRESENTATION:**

A 58-year-old man developed a chronic symptomatic seroma after open Rives–Stoppa repair for a midline incisional hernia. CT demonstrated an encapsulated fluid collection measuring approximately 10 × 7 cm dorsal to the rectus muscles, without signs of mesh infection. Because the symptoms persisted for 9 months despite repeated aspirations, laparoscopic fenestration through the posterior rectus sheath and retromuscular mesh was performed. Six fenestration openings were created to establish controlled communication between the seroma cavity and the peritoneal cavity. Percutaneous transfascial sutures were placed adjacent to the relatively larger defect to promote cavity collapse and reduce the potential risk of bowel incarceration. The postoperative course was uneventful. Follow-up MRI demonstrated substantial shrinkage of the chronic seroma cavity, with a small amount of asymptomatic omental protrusion through the mesh defect. Marked improvement in symptoms and QOL scores was observed after surgery.

**CONCLUSIONS:**

Laparoscopic fenestration through the posterior rectus sheath and retromuscular mesh may be a feasible, minimally invasive mesh-salvage option for selected patients with chronic symptomatic seroma after retromuscular incisional hernia repair in the absence of mesh infection.

## Abbreviations

IPMN
: intraductal papillary mucinous neoplasm
IPOM
: intraperitoneal onlay mesh

## INTRODUCTION

Postoperative seroma is a common complication after incisional hernia repair, particularly following retromuscular mesh placement. Although most seromas resolve spontaneously, some become chronic and symptomatic, resulting in persistent abdominal bulging, pain, and impaired QOL.^1)^ Management of chronic encapsulated seroma remains challenging because an optimal treatment strategy has not been established.^2)^

Repeated percutaneous aspiration may provide temporary symptom relief but carries a potential risk of secondary infection. In contrast, open anterior capsulectomy or open drainage may require wide exposure and dissection around the retromuscular mesh, potentially increasing the risks of secondary mesh infection, mesh destabilization, and recurrent incisional hernia.^3)^ Therefore, less invasive mesh-salvage strategies may be desirable for selected patients without evidence of mesh infection.

However, the literature regarding minimally invasive treatment of chronic seroma after retromuscular incisional hernia repair remains limited. Herein, I report a case of a chronic symptomatic seroma after Rives–Stoppa repair that was successfully managed by laparoscopic fenestration through the posterior rectus sheath and retromuscular mesh as a minimally invasive mesh-salvage approach.

## CASE PRESENTATION

A 58-year-old man developed a symptomatic chronic seroma after open retromuscular repair of a midline incisional hernia. His medical history included open abdominal aortic graft replacement for a ruptured abdominal aortic aneurysm, hypertension, IPMN, and cholelithiasis. He was an active smoker with a BMI of 29.2 kg/m^2^.

The patient underwent open Rives–Stoppa repair for a midline incisional hernia (European Hernia Society classification: M3, M4; W2) with a defect size of 14 × 5 cm, combined with cholecystectomy. After bilateral retro-rectus dissection, a 30 × 13 cm lightweight polypropylene mesh (Bard Soft Mesh, BD, Franklin Lakes, NJ, USA) was placed in the retromuscular space and secured to the posterior rectus sheath. No drain was placed during the initial repair.

**Fig. 1:**
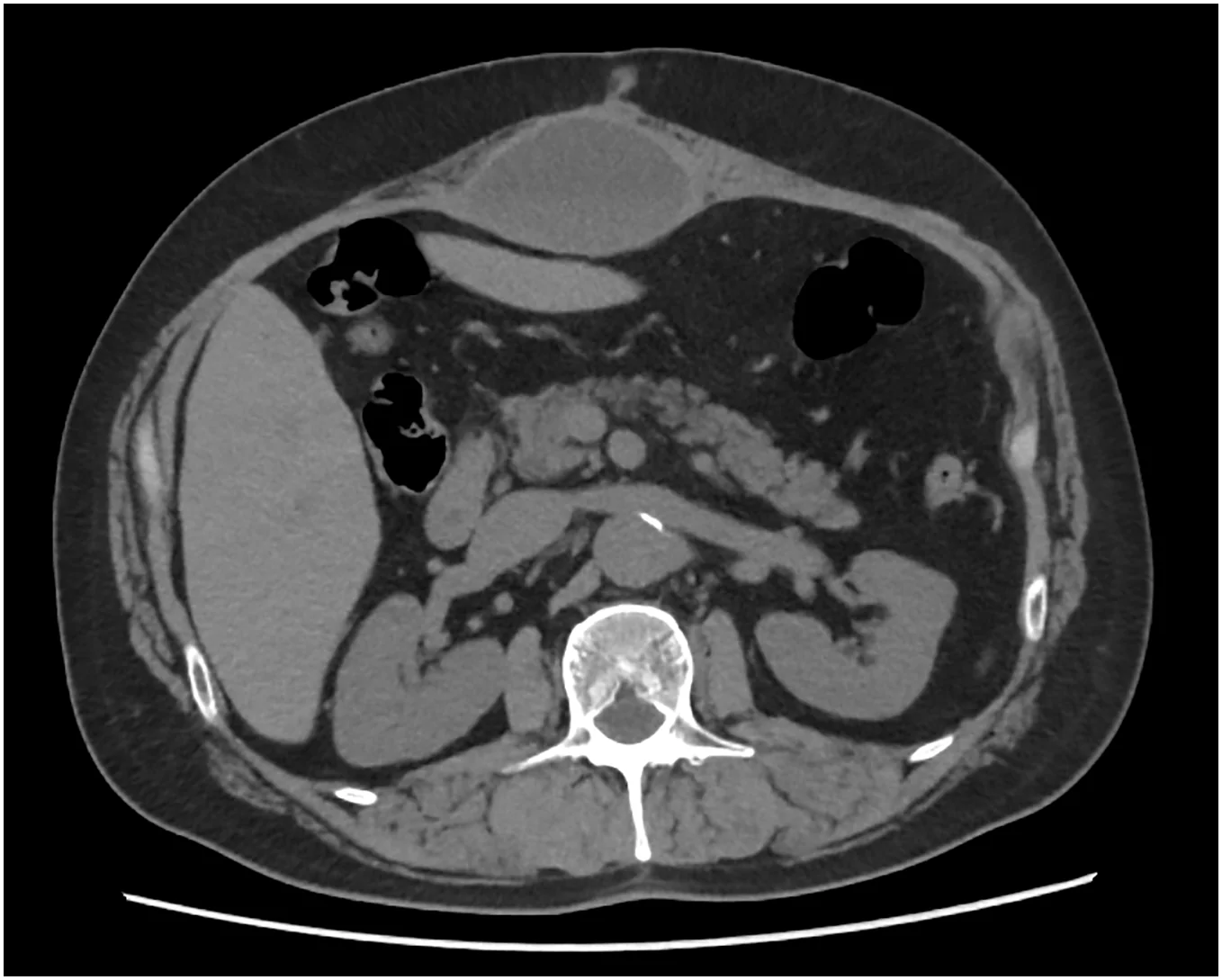
Preoperative CT findings of a chronic encapsulated seroma after Rives–Stoppa repair. Axial CT image obtained 6 months after retromuscular incisional hernia repair demonstrating a large encapsulated fluid collection interposed between the rectus muscle and the retromuscular mesh, without surrounding inflammatory enhancement or evidence of mesh infection.

Postoperatively, the patient developed persistent upper midline swelling and pain. Percutaneous aspiration was performed twice at a local clinic, yielding 70 and 80 mL of dark brown serous or hemorrhagic fluid, respectively. Bacterial cultures were negative; however, the symptoms persisted despite conservative management. CT demonstrated a chronic fluid collection measuring approximately 10 × 7 cm interposed between the rectus muscle and the retromuscular mesh, without surrounding enhancement or signs of mesh infection (**Fig. 1**). Because the lesion remained symptomatic 9 months after the index operation, surgical intervention was planned. Open anterior capsulectomy was considered potentially associated with secondary mesh contamination and subsequent mesh infection due to exposure of the retromuscular prosthesis. Therefore, a minimally invasive mesh-salvage approach was selected.

**Fig. 2:**
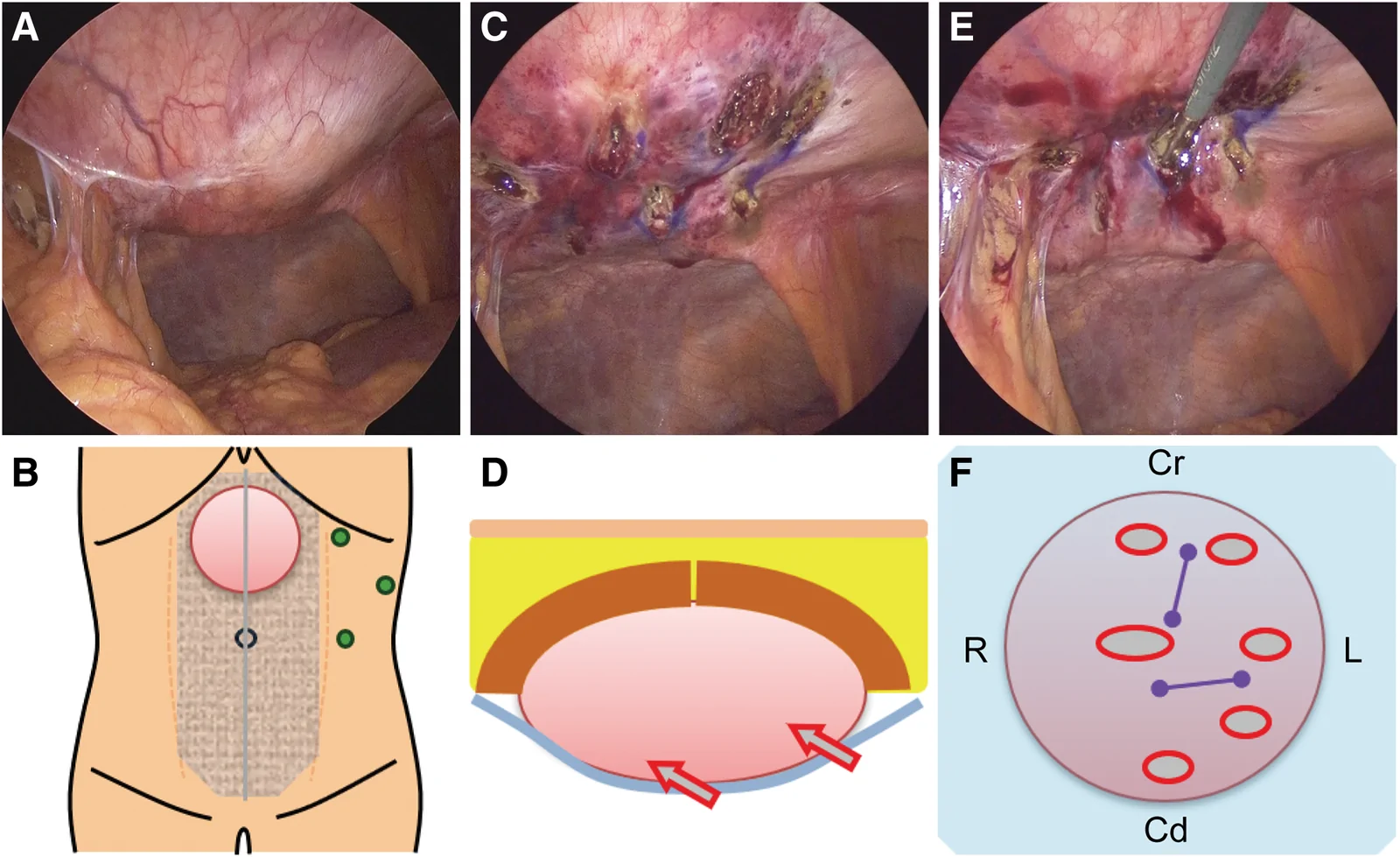
Intraoperative findings and schematic illustrations of laparoscopic fenestration through the retromuscular mesh. (**A**) Laparoscopic view demonstrating bulging of the chronic seroma cavity beneath the abdominal wall. (**B**) Schematic illustration of trocar placement and retromuscular mesh location. Green circles indicate 5-mm trocar sites. (**C**) Laparoscopic view after creation of multiple fenestration openings through the posterior rectus sheath and retromuscular mesh. (**D**) Schematic sectional illustration demonstrating internal drainage of the chronic seroma cavity through fenestration of the posterior rectus sheath and retromuscular mesh. (**E**) Instrument passage through the fenestration opening. The cavity wall was elevated using percutaneous transfascial sutures. (**F**) Axial schematic illustration of the fenestration sites (red) and percutaneous transfascial sutures (purple). Cd, caudal; Cr, cranial; L, left; R, right

Laparoscopic fenestration of the seroma cavity was performed (**Fig. 2A**). Intraoperatively, an approximately 8-cm bulging lesion corresponding to the seroma cavity was identified beneath the abdominal wall, without intraperitoneal inflammatory changes (**Fig. 2B**). After aspiration of dark reddish serous fluid for bacterial culture, which was subsequently negative, 6 fenestration openings were created through the fused peritoneum, posterior rectus sheath, retromuscular mesh, and seroma wall using ultrasonic shears, thereby establishing communication between the seroma cavity and the peritoneal cavity (**Fig. 2C**, **2D**). The fenestration openings were intended to remain smaller than 1 cm to minimize the risk of postoperative interparietal herniation through the mesh defects. However, 1 fenestration was enlarged to approximately 1.5 cm to facilitate laparoscopic instrumentation for the attempted excision of the fibrous capsule (**Fig. 2E**). Therefore, 2 percutaneous transfascial 3-0 polydioxanone sutures were placed adjacent to the defect to promote cavity collapse and reduce the potential risk of bowel incarceration (**Fig. 2F**). The cavity contained smooth, gray-white lining tissue, coagulated material, and orange-colored debris. Irrigation and drainage were performed without curettage. No drain was placed. The operative time was 231 minutes. Considerable time was spent attempting to excise the fibrous capsule under laparoscopic visualization.

The postoperative course was uneventful, and the patient was discharged on POD 2. Follow-up demonstrated marked improvement in symptoms and a progressive reduction in abdominal wall bulging.

**Fig. 3:**
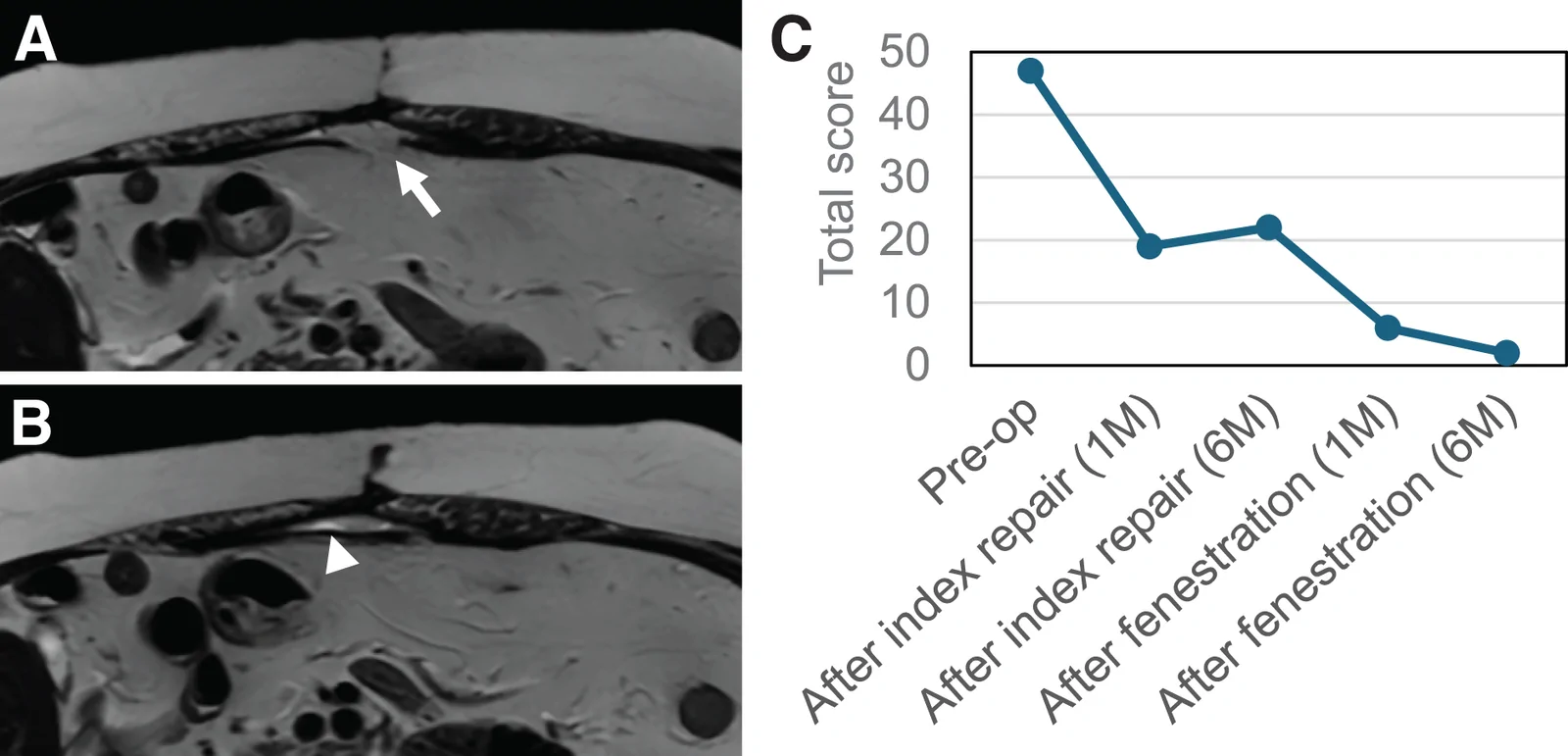
Postoperative MRI and QOL outcome after laparoscopic fenestration. (**A**) Postoperative axial T2-weighted MRI demonstrating omental protrusion through the mesh fenestration site (arrow) without bowel incarceration. (**B**) A small residual T2-high fluid space (arrowhead) adjacent to the omental interposition with substantial overall shrinkage of the chronic seroma cavity compared with the preoperative findings. (**C**) Transition of the total European Hernia Society QOL score demonstrating transient deterioration after the index repair and marked improvement after laparoscopic fenestration.

Approximately 6 months after laparoscopic fenestration, MRI performed during follow-up for IPMN demonstrated substantial shrinkage of the seroma cavity without evidence of recurrent incisional hernia or mesh infection. A small amount of omental protrusion through an approximately 1.5-cm mesh defect was observed, without associated bowel herniation or clinical symptoms (**Fig. 3A**). Although a small residual T2-high space remained (**Fig. 3B**), the patient experienced marked symptom improvement during follow-up (**Fig. 3C**).

## DISCUSSION

Chronic symptomatic seroma after retromuscular repair is uncommon, and no standardized treatment algorithm has been established.^2,4)^ In the present case, repeated percutaneous aspirations performed at a local clinic failed to achieve durable symptom control, whereas open anterior capsulectomy may require re-dissection of the reconstructed abdominal wall with disruption of the nonabsorbable fascial closure sutures and exposure of the retromuscular mesh, potentially increasing the risks of recurrent incisional hernia and mesh infection. Therefore, a minimally invasive laparoscopic mesh-salvage strategy was selected.

The key concept of the present procedure was internal drainage and decompression of the chronic seroma cavity through fenestration of the posterior rectus sheath and retromuscular mesh. By creating communication between the encapsulated cavity and the peritoneal cavity, reduction of fluid trapping, gradual cavity collapse, and adhesion of the cavity walls were expected. In contrast to open anterior approaches, this strategy avoided wide exposure of the retromuscular prosthesis.

An important technical consideration associated with this approach is balancing adequate drainage with preservation of abdominal wall integrity. First, the fenestration openings were intended to remain smaller than approximately 1 cm because complete tissue incorporation between the rectus muscle and the retromuscular mesh could not be assumed. This was considered important to preserve abdominal wall integrity while reducing the risks of recurrent incisional hernia and bowel incarceration. Second, the fenestrations were created primarily as transverse, slit-like incisions rather than large circular defects in order to minimize disruption of mesh tensile strength and preserve structural integrity of the retromuscular repair. In the present case, 1 fenestration was intentionally enlarged to approximately 1.5 cm to facilitate laparoscopic instrumentation for attempted excision of the fibrous capsule. Postoperative MRI demonstrated asymptomatic omental protrusion only through this enlarged fenestration, whereas no protrusion was observed through the remaining smaller fenestrations. Although transfascial sutures may help promote collapse of the residual cavity and reduction of dead space, omental protrusion through the relatively large fenestration may increase the risk of future traction pain or progressive enlargement of the hernia defect over time. Therefore, enlargement of the fenestration should be avoided whenever possible.

Another important anatomical consideration was the location of the fenestration sites. In the present case, the fenestration sites were located cranial to the transverse colon, where omental interposition rather than direct contact between the small bowel and the mesh defects was considered more likely. This anatomical relationship was considered favorable because it potentially reduced the risk of immediate small bowel incarceration through the fenestration sites. However, the same strategy may not be applicable to all patients. Particular caution is warranted in patients with a low BMI, seromas located in the lower abdomen, or situations in which the fenestration sites may come into direct contact with the small bowel.

An additional concern specific to this technique is the exposure of the cut edges of the polypropylene mesh to the peritoneal cavity. Such exposure is generally avoided because it may promote bowel adhesion to the exposed mesh edges, potentially leading to bowel erosion, fistula formation, mesh infection, or adhesion-related bowel obstruction. Accordingly, this technique should be reserved for carefully selected patients without mesh infection and used only when the anticipated benefits outweigh these potential risks. Although none of these complications developed during follow-up in the present case, longer-term clinical experience is required to establish the safety of this approach.

Various surgical strategies have been described for the management of chronic postoperative seroma after ventral hernia repair. Previous reports have included capsulectomy combined with argon beam scarification of the seroma wall to promote adhesion and obliteration of the residual cavity while emphasizing preservation of mesh integrity whenever possible.^3,5)^ Lehr and Schuricht also reported a minimally invasive approach using talc slurry seromadesis for a persistent postoperative seroma after incisional hernia repair.^6)^ These approaches primarily aim to eliminate the persistent encapsulated space.

In contrast, reports describing laparoscopic fenestration remain limited. A PubMed search identified only a few reports describing laparoscopic management of chronic postoperative seroma after IPOM repair, including laparoscopic seroscopy and internal drainage into the peritoneal cavity.^4,7)^ To the best of my knowledge, no previous report has described intentional fenestration through a retromuscular mesh for chronic seroma after Rives–Stoppa repair. Management of the pseudocapsule varied among the reported IPOM cases. One report described laparoscopic excision of the pseudocapsule, whereas another did not perform decortication. In the present case, complete excision of the fibrous capsule was not feasible because of the surrounding mesh, and the procedure was completed with fenestration and irrigation alone.

This report has several limitations. First, this was a single-case experience, and spontaneous improvement over time cannot be completely excluded. Second, the optimal number, size, and location of the fenestration openings remain unclear. Third, the long-term risk of interparietal herniation after mesh fenestration is unknown and requires careful follow-up.

## CONCLUSIONS

Laparoscopic fenestration through the posterior rectus sheath and retromuscular mesh may represent a feasible, minimally invasive mesh-salvage option in carefully selected patients with chronic symptomatic seroma after Rives–Stoppa incisional hernia repair in the absence of mesh infection.

## DECLARATIONS

### Availability of data and materials

All data supporting the conclusions of this article are included within the article.
